# Comparative proteomic analysis of colorectal cancer stem cells reveals potential biomarkers and altered pathways

**DOI:** 10.3389/fmolb.2026.1801265

**Published:** 2026-04-20

**Authors:** Ola J. Hussein, Lubna Therachiyil, Shahd M. Younis, Shaymaa Itani, Hanan H. Abunada, Issam Tout, Cristina Maccalli, Hesham M. Korashy

**Affiliations:** 1 Department of Pharmaceutical Sciences, College of Pharmacy, QU Health, Qatar University, Doha, Qatar; 2 Research Branch, Sidra Medicine, Doha, Qatar; 3 Translational Research Institute, Academic Health system, Hamad Medical Corporation, Doha, Qatar; 4 Biomedical Research Center, QU Health, Qatar University, Doha, Qatar; 5 Unit of Biotherapies, IRCCS Azienda Ospedaliera Metropolitana, Plesso Policlinico San Martino, Genova, Italy

**Keywords:** cancer stem cells, cancer-initiating cells, colorectal cancer, LC-MS/MS, proteomics, spheroids

## Abstract

**Background:**

Colorectal cancer (CRC) initiating/stem cells (CICs/CSCs) represent a rare tumor subpopulation with self-renewal capacity that drives tumor progression, recurrence, therapeutic resistance, and immune evasion. Despite extensive efforts to define CSCs using surface and functional markers, no universally accepted marker exists for CSC isolation and enrichment. Moreover, the molecular mechanisms underlying CSC-associated phenotypes remain incompletely characterized, highlighting the need for unbiased proteome-wide molecular profiling to better define CSC states and identify candidate biomarkers and therapeutic targets.

**Methods:**

CSC-enriched spheroids were generated from two colorectal cancer cell lines (SW620 and HCT-116) using three-dimensional, serum-free culture conditions and compared with their corresponding parental adherent cells. Comparative proteomic profiling was performed using mass spectrometry-based label-free shotgun proteomics. Differentially abundant proteins were analyzed using Ingenuity Pathway Analysis (IPA) to identify overrepresented canonical pathways and predict upstream regulators. Selected differentially abundant proteins and predicted upstream regulators were validated by RT-qPCR and/or Western blotting.

**Results:**

Comparative proteomic profiling showed that CSC-enriched spheroids shared convergent pathway-level alterations despite cell line-specific differences in individual protein abundance. IPA pathway and functional analyses predicted activation of metabolic reprogramming, invasion, and hypoxia adaptation, along with predicted suppression of apoptotic pathways. Notably, HMGCS1, a key mevalonate-pathway enzyme, was strongly upregulated at both mRNA and protein levels in CSCs from both cell lines. MYC, MLXIPL, EGF/EGFR, VEGFA, and HIF-related signaling were among the top predicted upstream regulators shaping these alterations. In addition, altered expression of proteins involved in immunosuppressive signaling was observed in CSC-enriched spheroids, with TGF-β signaling emerging as a prominently activated upstream regulator, potentially contributing to CSC-associated epithelial-mesenchymal transition and immunomodulation.

**Conclusion:**

In summary, this study provides a better understanding of key dysregulated pathways and proteins in CRC CSCs, highlighting potential biomarkers and regulatory programs with relevance to stemness, immune modulation, and therapeutic resistance.

## 1 Introduction

Colorectal cancer (CRC) is the third most commonly diagnosed malignancy and the second leading cause of cancer-related mortality worldwide (Global Cancer Observatory GCO, 2025). Despite major advances in early screening and the introduction of targeted therapies that have improved overall survival (OS), a substantial proportion of patients exhibit either primary resistance or develop acquired resistance during treatment, ultimately progressing to advanced, metastatic disease (SEER, 2025; Tang et al., 2023). In contrast to localized CRC, metastatic CRC is associated with a poor prognosis, with an approximate 5-year survival rate of about 16.2% (SEER, 2025). Accordingly, identifying molecular drivers of tumor progression and therapeutic resistance remains essential to improving long-term clinical outcomes.

Tumors are composed of heterogeneous cell populations with distinct phenotypic and functional properties. Within this diversity, cancer stem cells (CSCs), also referred to as cancer stem-like cells (CSLCs) or cancer-initiating cells (CICs), represent a minor subpopulation endowed with “stemness” features, including self-renewal capacity, the ability to enter quiescence, and pluripotency (Maccalli et al., 2018; Lee H. et al., 2025). CSCs are increasingly recognized as key contributors to tumor initiation, metastasis, and resistance to conventional therapies (Jiang et al., 2025; Chu et al., 2024). Moreover, emerging evidence indicates that CSCs may facilitate immune evasion and influence responses to immunotherapies (Hussein et al., 2025; Ravindran et al., 2019; Gupta et al., 2023). Together, these findings underscore the importance of elucidating CSC-associated molecular programs as a prerequisite for developing more effective and durable therapeutic strategies and biomarkers.

Despite their recognized biological and clinical relevance, CSCs remain challenging to define and isolate due to their low abundance in tumor tissue, pronounced heterogeneity, and phenotypic plasticity. Although multiple surface markers (e.g., CD44, CD133, EpCAM, CD166, and CD44v6), as well as intracellular and functional markers (e.g., ALDH and LGR5), have been used to identify and enrich CRC stem-like populations (Pashirzad et al., 2022; Maccalli and De Maria, 2015), these markers exhibit limited specificity as they are also expressed by normal cells and non-CSCs, often at lower levels (Chu et al., 2024). Furthermore, the expression of these markers may vary depending on proliferative and differentiation states, reflecting CSC plasticity and substantial intra- and inter-tumoral heterogeneity (Tout et al., 2025). These limitations complicate the isolation of well-defined CSC populations and hinder comprehensive molecular characterization using marker-based strategies alone.

To address these challenges, cancer cell lines have been shown to provide an alternative, reproducible source for CSC research. Three-dimensional (3D) spheroid culture under non-adherent, serum-free conditions is widely used to enrich for cancer stem or stem-like populations by exploiting key CSC-associated properties, such as resistance to anoikis, stress tolerance, and self-renewal capacity (Weiswald et al., 2015). Large-scale proteomic and proteogenomic studies have identified dysregulated signaling pathways, biomarkers and drug targets (Vasaikar et al., 2019). In our recent work, we successfully enriched CSCs from four CRC cell lines, with HCT-116- and SW620-derived spheroids exhibiting the highest self-renewal potential, as evidenced by sustained spheroid formation across multiple passages (Hussein et al., 2026). In order to deepen the understanding of the molecular features of CSCs, a proteomic approach was employed.

Recent advances in mass spectrometry–based proteomics have significantly expanded our understanding of CRC biology (Zhang B. et al., 2014). Large-scale proteomic and proteogenomic studies have identified dysregulated signaling pathways, biomarkers and drug targets (Vasaikar et al., 2019; Wiśniewski et al., 2015). Importantly, proteomic profiling provides complementary insights beyond transcriptomic analyses by capturing post-transcriptional regulation and functional pathway activity that cannot be reliably inferred from gene expression alone. Nevertheless, proteome-level alterations distinguishing colorectal differentiated tumor cells from their stem-like counterparts remain poorly characterized. In this study, we performed a comparative proteomic analysis of colorectal CSC-enriched spheroids vs. parental bulk cancer cells to gain deeper insights into differentially regulated proteins and pathways, with the aim of identifying CSC-associated molecular signatures that may guide biomarker discovery and therapeutic targeting.

## 2 Materials and methods

### 2.1 Cell culture of cancer cells

Two colorectal cancer cell lines, HCT-116, that has epithelial morphology, and SW620, that has fibroblast-like morphology, were obtained from the American Type Culture Collection (ATCC). Cells were cultured in high-glucose Dulbecco’s Modified Eagle’s Medium (DMEM) containing GlutaMAX (Gibco; cat. 31,966–047), supplemented with 10% heat-inactivated fetal bovine serum (FBS) and 1% Antibiotic-Antimycotic (Gibco; cat. 15,240–062) and maintained at 37 °C in a humidified incubator with 5% CO_2_.

### 2.2 CSC-enriched spheroid culture

To enrich for CSC populations, bulk HCT-116 and SW620 cells were harvested and seeded at a density of 50,000 cells/mL in ultra-low-attachment T-75 flasks (Nunclon Sphera, Thermo Fisher Scientific). Cells were cultured in serum-free DMEM/F12 (StemFlex medium; Gibco; cat. A3349401) supplemented with 1× StemFlex supplement and 1× Antibiotic-Antimycotic. Spheroids were maintained through sequential passaging to further enrich for CSCs. Briefly, spheroids were collected by gravitational sedimentation for 10 min, enzymatically dissociated into single cells using 1× TrypLE Express (Gibco, 12,605–028), and mechanically by gentle pipetting. The resulting single-cell suspension was then reseeded at the same density under the same culture conditions (Rybak et al., 2011; Mukherjee et al., 2021).

### 2.3 Protein extraction for the whole proteome analysis

Protein extraction was performed as previously described (Therachiyil et al., 2024; Dhulkifle et al., 2024). Briefly, cells were washed with ice-cold PBS, and total proteins were extracted using RIPA lysis buffer containing 1× Halt™ Protease Inhibitor Cocktail (Thermo Scientific; cat. 78429). Protein concentrations were determined using the Pierce™ Rapid Gold BCA Protein Assay Kit (Thermo Scientific; cat. A53225) following the manufacturer’s instructions.

### 2.4 Sample preparation for mass spectrometry analysis

Proteins from three independent replicates for each cell line were reduced with 5 mM dithiothreitol for 40 min at RT and alkylated with 10 mM iodoacetamide for 30 min at RT in the dark. Samples were then digested overnight at 37 °C in ABC buffer using Mass Spec Grade Trypsin/Lys-C Mix (Promega; Madison, WI, United States of America; 12.5 ng/μL) at an enzyme-to-protein ratio of 1:25. Digestion was quenched by acidifying the reaction mixture with 1% formic acid (FA). Peptides were subsequently desalted and concentrated by solid-phase extraction using Bond Elut C18 cartridges (Agilent Technologies) and eluted with stepwise gradients of acetonitrile (ACN) in 0.1% FA. The eluate was dried in a SpeedVac concentrator to obtain a peptide pellet, reconstituted in 2% ACN containing 0.1% FA, and subsequently analyzed by mass spectrometry (Therachiyil et al., 2024; Rappsilber et al., 2007).

### 2.5 Liquid chromatography-tandem mass spectrometry (LC-MS/MS) analysis

Trapped ion mobility mass spectrometry (TIMS) analyses were performed on a timsTOF Pro mass spectrometer (Bruker Daltonics, Germany) coupled to a nanoElute nano-liquid chromatography system (Bruker Daltonics). For each run, 10 μL of peptide solution corresponding to 2 μg of total peptides were injected into the LC-MS system. Desalted peptides were separated by reversed-phase chromatography on a C18 column with an integrated CaptiveSpray emitter (25 cm × 75 μm, 1.6 µm; IonOpticks, Australia) at a flow rate of 400 nL/min using mobile phase A (ultrapure water containing 0.1% FA) and mobile phase B (ACN containing 0.1% FA). The gradient was run from 2% to 80% B over 110 min, with a total run time of 120 min. MS data were acquired in PASEF data-dependent acquisition (PASEF-DDA) mode, selecting the top 10 most abundant precursor ions from each full MS survey scan (m/z 400–1800) for fragmentation and MS/MS analysis. Precursors with a charged state of +1 were rejected, and the dynamic exclusion duration was set to 25 s.

### 2.6 MS and MS/MS data processing and analysis

Raw MS/MS data were processed in MaxQuant (v2.1.4.0) using the integrated Andromeda search engine, following the standard workflow (Tyanova et al., 2016a; Cox et al., 2011). Protein identification was performed by searching against the UniProtKB/Swiss-Prot human reference proteome (UP000005640_9606) database. Methionine oxidation was set as a variable modification, whereas carbamidomethylation of cysteine was defined as a fixed modification. Mass tolerances were set according to instrument-specific default settings in MaxQuant (first search 20 ppm; main search 10 ppm; MS/MS tolerance default). A maximum of one missed cleavage was allowed for tryptic digestion. Peptide-spectrum matches and protein identifications were filtered at a 1% false discovery rate based on a reverse-sequence decoy database.

Label-free quantification (LFQ) was performed using the MaxLFQ algorithm in MaxQuant, as previously described (Cox et al., 2014) with an LFQ minimum ratio count of 2. Retention-time alignment and the match-between-runs feature were enabled (match time window 0.7 min; alignment time window 20 min, default). Protein abundance was inferred from LFQ intensity values.

Downstream data processing was performed in Perseus (v2.0.7.0) (Therachiyil et al., 2024; Tyanova et al., 2016b). Proteins annotated as potential contaminants, reverse-sequence matches, and those identified only by site were excluded. LFQ intensities from the MaxQuant were Log_2_-transformed, and proteins consistently quantified in at least two biological replicates of at least one experimental condition were retained for subsequent comparative analysis. Missing values were imputed in Perseus by normal distribution according to default settings (width = 0.3; downshift = 1.8). Differential protein abundance was assessed using a two-tailed Student’s t-test with a permutation-based false discovery rate control set to 5% and absolute Log2 fold-change (Log_2_FC|) ≥ 1.5. Principal component analysis (PCA) was performed to evaluate the reproducibility and clustering of biological replicates. The raw mass spectrometry proteomics data have been deposited in the PRIDE repository via the ProteomeXchange Consortium under the dataset identifier PXD075150. A complete list of differentially abundant proteins is provided in the Supplementary Material.

### 2.7 Ingenuity Pathway Analysis

Ingenuity Pathway Analysis (IPA; QIAGEN) was used for exploratory functional enrichment and pathway analysis. Lists of proteins, together with their Log_2_FC values and p-values, were uploaded into the IPA application and analyzed. Proteins with a p-value <0.05 for SW620 and adjusted p-value <0.05 for HCT-116 together with an effect-size cutoff of |Log_2_FC| ≥ 1 were included in the IPA analysis. This threshold was used to retain a sufficient number of proteins for pathway enrichment analysis. IPA was used to identify over-represented canonical pathways, upstream regulators, and disease and biological function annotations based on the Ingenuity Knowledge Base, which is curated from published experimental literature. For canonical pathway enrichment, IPA calculates statistical significance using a right-tailed Fisher’s exact test, and pathway significance is presented as -Log_10_ (p-value). Predicted pathway activation or inhibition was assessed using the IPA activation z-score algorithm, which incorporates the direction of protein abundance changes.

### 2.8 Quantitative real‐time PCR (RT-qPCR)

Total RNA was extracted from cells using PureLink™ RNA Mini Kit (Invitrogen, cat. 12183025) according to the manufacturer’s instructions. RNA purity and concentration were determined by NanoDrop™ 8000 Spectrophotometer. Reverse transcription was performed using High-Capacity cDNA Reverse Transcription Kit (Applied Biosystems, cat. 4374966). qPCR was performed on a QuantStudio™ 12K Flex Real-Time PCR System using PowerUp™ SYBR™ Green Master Mix (Applied Biosystems, cat. A25742). Relative gene expression was calculated using the comparative ΔΔCt method, with GAPDH as the reference gene. Primer sequences are provided in Supplementary Table S1.

### 2.9 Western blot analysis

Proteins (30 μg) were separated by sodium dodecyl sulfate-polyacrylamide gel electrophoresis (SDS-PAGE) followed by immunoblotting as previously described (Therachiyil et al., 2022). Bands were visualized using SuperSignal™ West Pico PLUS Chemiluminescent Substrate (Thermo Scientific, cat. 34580) and imaged on a ChemiDoc Imaging System (Bio-Rad). Densitometry analysis was performed using ImageJ (https://imagej.net/ij/). The protein bands in the figures are a composite of different blots and are representative blots for the indicated proteins and the loading control. Lists of the antibodies used are provided in Supplementary Table S2.

### 2.10 Statistical analysis

For proteomics, differential protein abundance was assessed using a two-tailed Student’s t-test with a permutation-based FDR of 5% to control for multiple testing in Perseus. For RT-qPCR and Western blot assays, significance between two groups was evaluated using an unpaired two-tailed Student’s t-test by GraphPad Prism (version 10) with p < 0.05 considered statistically significant. Data are presented as mean ± SEM from at least three independent experiments.

## 3 Results

### 3.1 LC-MS/MS analysis identified differentially abundant proteins in colorectal CSCs

To compare the global protein abundance profiles between CSC-enriched spheroids and their corresponding adherent parental colorectal cancer cells (HCT-116 and SW620), label-free quantitative proteomic analysis was performed.

**FIGURE 1:**
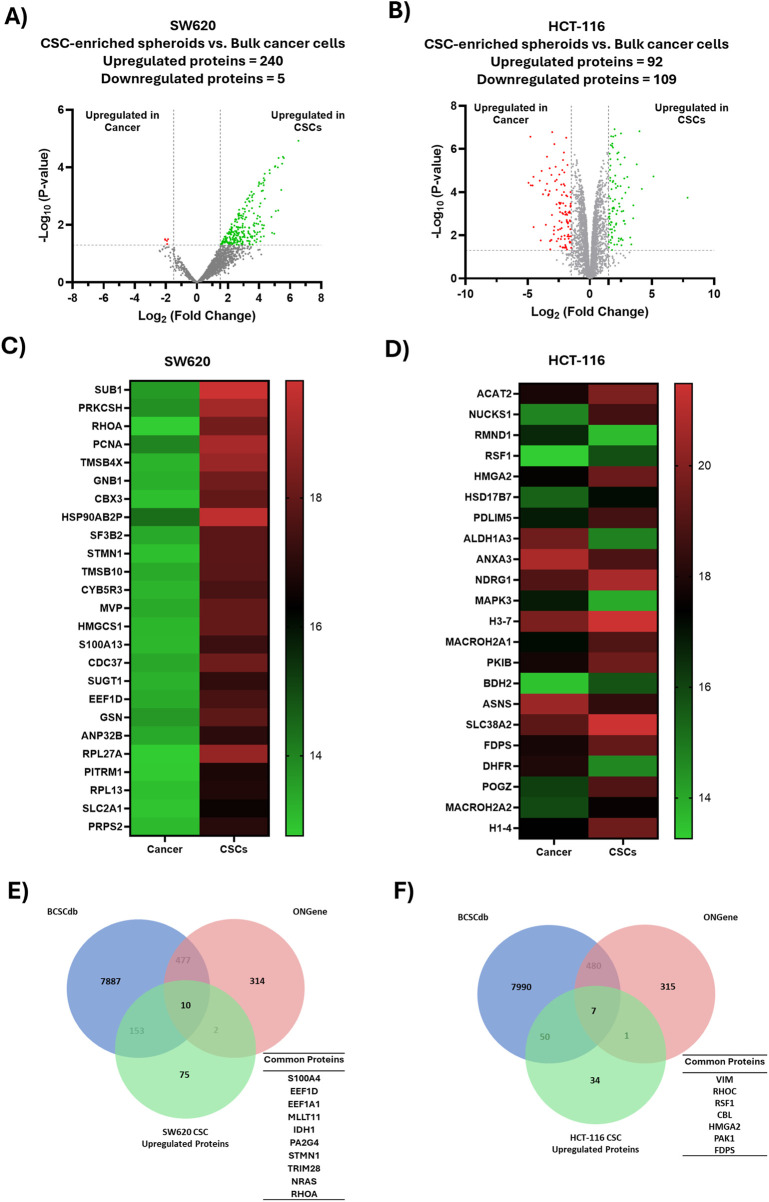
Differential proteomic profiling of colorectal CSCs. **(A,B)** Volcano plots of differentially abundant proteins between spheroid-enriched CSCs and bulk cancer cells from SW620 **(A)** and HCT-116 **(B)** cell lines. Log_2_FC (x-axis) is plotted against -Log_10_ (p-value) (y-axis). Each point represents an individual protein and is color-coded in green or red when up- or downregulated in CSCs, respectively, and in gray when non-significant. Differentially abundant proteins were defined using a statistical threshold of p < 0.05 together with an effect-size cutoff of |Log_2_FC| ≥ 1.5. **(C,D)** Heatmaps showing differentially abundant proteins in CSCs derived from SW620 **(C)** and HCT-116 **(D)**. **(E,F)** Venn diagrams showing the overlap between CSC-upregulated proteins and proteins listed in the ONGene oncogene database and the BCSCdb CSC biomarker database. Shared CSC-associated proteins for each comparison are listed.

Using a significance threshold of p < 0.05 together with an effect-size cutoff of |Log_2_FC| ≥ 1.5, SW620 CSCs exhibited 245 differentially abundant proteins (240 upregulated and 5 downregulated), whereas HCT-116 CSCs exhibited 201 differentially abundant proteins (92 upregulated and 109 downregulated) relative to their parental cancer cells (Figures 1A,B). Significant differentially abundant proteins between CSC-enriched spheroids and bulk cancer cells are visualized in the heatmaps (Figures 1C,D). Additionally, proteins showing the largest magnitude of change (|Log_2_FC|) are listed in Tables 1,2.

**TABLE 1:** Differentially expressed proteins with the largest magnitude of change in SW620 CSC-enriched spheroids versus bulk cancer cells.

Protein ID	Gene name	Protein name	Log_2_ FC	Adjusted p-value	p-value
P53999	SUB1	Activated RNA polymerase II transcriptional coactivator p15	6.53	0.0177	<0.0001
P12004	PCNA	Proliferating cell nuclear antigen	5.59	0.0177	<0.0001
P14314	PRKCSH	Glucosidase 2 subunit beta	5.53	0.0177	<0.0001
P62328	TMSB4X	Thymosin beta-4; hemo regulatory peptide AcSDKP	5.48	0.0187	0.0001
P46776	RPL27A	Large ribosomal subunit protein uL15	5.42	0.0431	0.0006
P61586	RHOA	Transforming protein RhoA	5.25	0.0177	<0.0001
Q58FF8	HSP90AB2P	Putative heat shock protein HSP 90-beta 2	5.20	0.0187	0.0001
P62873	GNB1	Guanine nucleotide-binding protein G(I)/G(S)/G(T) subunit beta-1	5.04	0.0187	0.0001
Q13185	CBX3	Chromo box protein homolog 3	5.01	0.0187	0.0001
P16949	STMN1	Stathmin	4.69	0.0225	0.0002
Q13435	SF3B2	Splicing factor 3B subunit 2	4.67	0.0204	0.0001
P63313	TMSB10	Thymosin beta-10	4.36	0.0225	0.0002
Q14764	MVP	Major vault protein	4.35	0.0304	0.0003
Q01581	HMGCS1	Hydroxy methyl glutaryl-CoA synthase, cytoplasmic	4.33	0.0304	0.0003
P00387	CYB5R3	NADH-cytochrome b5 reductase 3	4.29	0.0294	0.0002
P06396	GSN	Gelsolin	4.29	0.0327	0.0004
Q16543	CDC37	Hsp90 co-chaperone Cdc37; Hsp90 co-chaperone Cdc37, N-terminally processed	4.26	0.0311	0.0003
Q5JRX3	PITRM1	Presequence protease, mitochondrial	4.24	0.0431	0.0006
P26373	RPL13	Large ribosomal subunit protein eL13	4.16	0.0431	0.0007
Q9Y2Z0	SUGT1	Protein SGT1 homolog	4.13	0.0311	0.0004

Top 20 differentially expressed proteins, demonstrating the highest intensity of change in SW620 CSC-enriched spheroids compared with bulk cancer cells. The proteomic dataset was filtered to include proteins meeting p < 0.01 and adjusted p < 0.05 and absolute Log_2_FC > 1.5. The 20 proteins with the greatest absolute magnitude of change were selected and ranked according to their Log_2_FC., The table reports UniProt protein ID, gene symbol, protein name, Log_2_FC, adjusted p-value, and p-value.

**TABLE 2:** Differentially expressed proteins with the largest magnitude of change in HCT-116 CSC-enriched spheroids versus bulk cancer cells.

Protein ID	Gene name	Protein name	Log_2_ FC	Adjusted p-value	p-value
P08670	VIM	Vimentin	7.86	0.0027	0.0002
P07203	GPX1	Glutathione peroxidase 1	5.11	0.0010	<0.0001
P08134	RHOC	Rho-related GTP-binding protein RhoC	4.17	0.0017	0.0001
Q9H1E3	NUCKS1	Nuclear ubiquitous casein and cyclin-dependent kinase substrate 1	3.98	0.0001	<0.0001
Q8WVX9	FAR1	Fatty acyl-CoA reductase 1	3.77	0.0005	<0.0001
Q9Y394	DHRS7	Dehydrogenase/reductase SDR family member 7	3.50	0.0041	0.0004
Q4V328	GRIPAP1	GRIP1-associated protein 1; GRASP-1 C-terminal chain	3.49	0.0092	0.0016
P00374	DHFR	Dihydrofolate reductase	−3.45	0.0003	<0.0001
Q99988	GDF15	Growth/differentiation factor 15	−3.45	0.0014	<0.0001
Q9Y276	BCS1L	Mitochondrial chaperone BCS1	−3.55	0.0143	0.0033
Q13257	MAD2L1	Mitotic spindle assembly checkpoint protein MAD2A	−3.58	0.0019	0.0001
Q6P1X6	C8orf82	UPF0598 protein C8orf82	−3.77	0.0023	0.0001
Q92572	AP3S1	AP-3 complex subunit sigma-1	−3.88	0.0007	<0.0001
Q9NPH2	ISYNA1	Inositol-3-phosphate synthase 1	−4.03	0.0012	<0.0001
Q96TA2	YME1L1	ATP-dependent zinc metalloprotease YME1L1	−4.52	0.0168	0.0041
Q9UEW8	STK39	STE20/SPS1-related proline-alanine-rich protein kinase	−4.53	0.0010	<0.0001
Q9H4G0	EPB41L1	Band 4.1-like protein 1	−4.61	0.0015	<0.0001
Q9NY61	AATF	Protein AATF	−4.75	0.0015	<0.0001
P47895	ALDH1A3	Retinaldehyde dehydrogenase 3	−4.78	0.0001	<0.0001
O00483	NDUFA4	Cytochrome c oxidase subunit NDUFA4	−4.94	0.0013	<0.0001

Top 20 differentially abundant proteins showing the largest magnitude of change in HCT-116 CSC-enriched spheroids compared with bulk cancer cells. The proteomic dataset was filtered to include proteins meeting p < 0.01 and adjusted p < 0.05 and an effect-size cutoff of |Log_2_FC| ≥ 1.5. The 20 proteins with the greatest absolute magnitude of change were selected and ranked according to their Log_2_(FC). The table reports UniProt protein ID, gene symbol, protein name, Log_2_(FC), adjusted p-value, and p-value.

Among key upregulated proteins (adjusted p < 0.05) in SW620 CSC-enriched spheroids were SUB1, PCNA, PRKCSH, and TMSB4X, whereas VIM, GPX1, RHOC, and NUCKS1 were among the upregulated proteins in HCT-116 CSC-enriched spheroids. Notably, HMGCS1, ACAT2, CTSD, LAMP1 and CORO1B were among the proteins commonly upregulated in both cell lines (p < 0.05). Given that CSC phenotypes are frequently associated with oncogenic signaling, CSC-upregulated proteins were matched against entries in the ONGene oncogene database and with CSC-related biomarkers curated in BCSCdb. Figures 1E,F summarize these overlaps, highlighting a subset of CSC-upregulated proteins with prior evidence of oncogenic and CSC relevance (e.g., VIM, NRAS, and S100A4).

### 3.2 Functional annotation and pathway enrichment of CSC-associated proteins

IPA was employed to investigate the biological relevance of proteins differentially abundant between CSC-enriched spheroids and bulk cancer cells, with a focus on canonical pathways and associated disease and functions.

**FIGURE 2:**
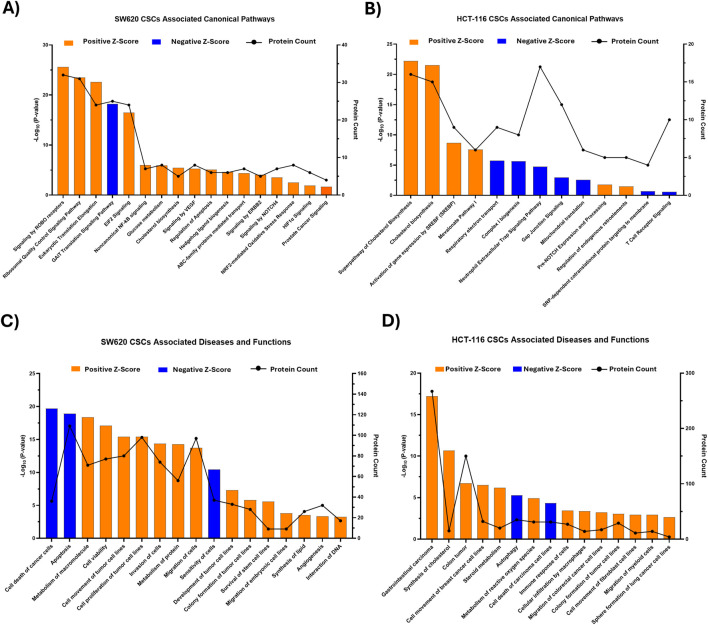
Ingenuity Pathway Analysis (IPA)-based functional and pathway enrichment analysis of colorectal cancer stem cell (CSC)-associated proteomic profiles. Spheroid-enriched CSCs derived from SW620 and HCT-116 cells were compared with the corresponding bulk (parental) cancer cells from the same cell line. **(A,B)** Top enriched canonical pathways associated with SW620 CSCs **(A)** and HCT-116 CSCs **(B)**. **(C,D)** Top enriched diseases and functions associated with SW620 CSCs **(C)** and HCT-116 CSCs **(D)**. Bars represent pathway/functional enrichment significance shown as -Log_10_(P-value). Bar color indicates IPA activation z-score: orange = positive z-score (predicted activation) and blue = negative z-score (predicted inhibition). The black line indicates the number of proteins mapped to each pathway/function.

As shown in Figure 2A, the top enriched canonical pathways in SW620 CSCs that were predicted to be activated were primarily related to translational regulation and stress-adaptive responses (e.g., Eukaryotic Initiation Factor 2 [eIF2] signaling, ribosomal quality control, eukaryotic translation elongation), metabolic reprogramming (e.g., cholesterol biosynthesis) together with hypoxia-associated signaling (e.g., HIF-1α signaling) and oncogenic signaling (e.g., VEGF signaling and Erb-B2 receptor tyrosine kinase 2 [ERBB2/HER2] signaling). In HCT-116 CSCs, canonical pathways predicted to be activated were predominantly linked to metabolic reprogramming (e.g., super pathway of cholesterol biosynthesis, cholesterol biosynthesis I, SREBF-mediated gene expression, and the mevalonate pathway) (Figure 2B). Consistent with these pathway-level changes, IPA disease and function analysis indicated that CSC-associated proteins in both cell lines were strongly linked to cancer-relevant functional programs (Figures 2C,D). Overall, the top activated functions were related to cell survival/viability, proliferation, cellular movement, migration, invasion and carcinoma/tumor development, together with metabolic processes. Conversely, the top inhibited functions were predominantly associated with apoptosis, cell death, and cellular sensitivity, consistent with a CSC phenotype favoring tumor persistence and progression. An illustrative summary generated using IPA is provided in Supplementary Figures S1-S3

### 3.3 Functional categorization and mRNA-level validation of CSC-associated proteins

**FIGURE 3:**
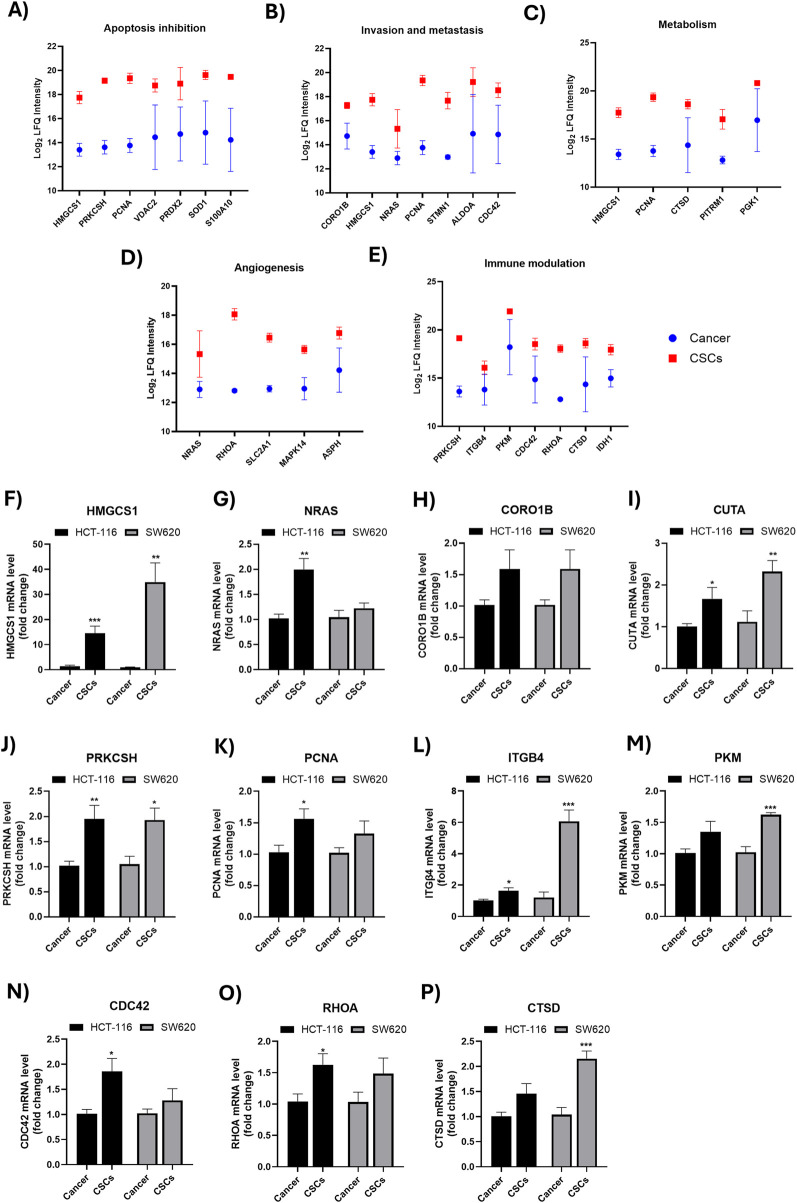
Functional categorization and validation of selected differentially abundant proteins in colorectal CSCs. **(A–E)** Plots showing Log_2_ LFQ intensity (mean ± SEM) of selected proteins in SW620-derived CSC-enriched spheroids (red) vs. bulk SW620 cancer cells (blue), grouped by their reported involvement in **(A)** Apoptosis inhibition, **(B)** invasion and metastasis, **(C)** metabolism, **(D)** angiogenesis and **(E)** immune modulation. **(F–P)** RT-qPCR validation of selected targets in SW620 and HCT-116 CSC-enriched spheroids vs. their corresponding bulk cancer cells. mRNA levels were normalized to the expression of the housekeeping gene GAPDH. Data are presented as mean ± SEM from at least three independent experiments relative to bulk cancer cells of the same cell line. *P < 0.05, **P < 0.01, ***P < 0.001.

The highly expressed proteins that were significantly altered in CSC-enriched spheroids were further categorized according to their predominant biological functions, including apoptosis inhibition, invasion and metastasis, metabolism, angiogenesis, and immune modulation, as illustrated in Figures 3A–E; Supplementary Tables S3-S5. To further validate the differential expression of CSC-associated markers, the mRNA expression of selected functionally relevant proteins was quantified by RT-qPCR. Figures 3F–P, representing key functional categories, including apoptosis inhibition (*PRKCSH*, *HMGCS1*, *PCNA*), invasion and metastasis (*CORO1B*, *HMGCS1*, *NRAS*), angiogenesis (*NRAS*, *RHOA*), metabolic reprogramming (*HMGCS1*, *PCNA*, *CTSD*) and immune modulation (*ITGB4*, *PKM*, *CDC42*, *RHOA*, *CTSD*), were upregulated in CSCs, that were consistent with the proteomic results. Remarkably, *HMGCS1* was significantly upregulated at the mRNA level in the CSCs of both SW620 and HCT-116 cells (34.9-fold and 14.6-fold, respectively), consistent with corresponding increases at the protein level (20.2-fold and 11.1-fold, respectively) relative to bulk cancer cells.

### 3.4 Upstream regulator analysis of CSC-associated proteomic signatures

**FIGURE 4:**
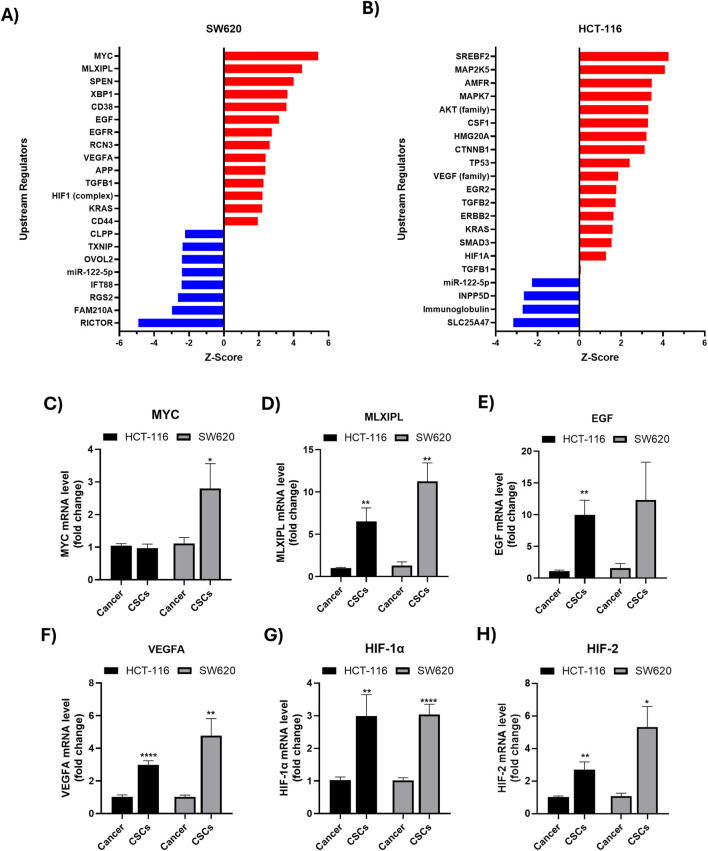
Predicted upstream regulator analysis of differentially expressed proteins in colorectal CSCs. **(A,B)** Ingenuity Pathway Analysis (IPA) Upstream Regulator Analysis based on differentially abundant proteins in **(A)** SW620 and **(B)** HCT-116 CSC-enriched spheroids compared with the corresponding bulk (parental) cancer cells. Bars represent the IPA activation z-score, where red indicates predicted activation (positive z-score) and blue indicates predicted inhibition (negative z-score). **(C–H)** RT-qPCR validation of selected upstream regulators in SW620 and HCT-116 CSC-enriched spheroids versus their corresponding bulk cancer cells. mRNA levels were normalized to the housekeeping gene GAPDH. Data are presented as mean ± SEM from at least three independent experiments relative to bulk cancer cells of the same cell line. *P < 0.05, **P < 0.01, ***P < 0.001, ****P < 0.0001.

Upstream regulator analysis was performed using IPA to infer potential molecular drivers underlying the CSC-associated proteomic changes based on the coordinated expression patterns of downstream target proteins. In SW620 CSC-enriched spheroids, IPA predicted activation of multiple regulators implicated in stemness maintenance (MYC, CD44), metabolic reprogramming (MLXIPL, XBP1), hypoxia adaptation (HIF1 complex), and oncogenic signaling (EGF/EGFR-related signaling, VEGFA, KRAS). In contrast, a subset of regulators linked to tumor-suppressive or differentiation-related programs, such as TXNIP, OVOL2, and miR-122-5p, were predicted to be inhibited (Figure 4A). Similarly, in HCT-116 CSC-enriched spheroids, IPA predicted activation of upstream regulators linked to growth factor signaling (CSF1), hypoxia adaptation (HIF-related signaling), TGF-β signaling (SMAD3/TGF-β-associated regulators), metabolic regulation (SREBF2) and oncogenic pathways (KRAS) (Figure 4B). To experimentally validate these *in silico* predictions, RT-qPCR analysis was performed. CSC-enriched spheroids from both cell lines exhibited significantly increased mRNA expression of selected predicted upstream regulators, including *MYC*, *MLXIPL*, *EGF*, *VEGFA*, *HIF-1α*, and *HIF-2α*, compared with bulk cancer cells (Figures 4C–H). Together, these findings support the involvement of these regulators in shaping CSC-associated molecular programs.

### 3.5 Activation of TGF-β signaling in colorectal CSC-enriched spheroids

**FIGURE 5:**
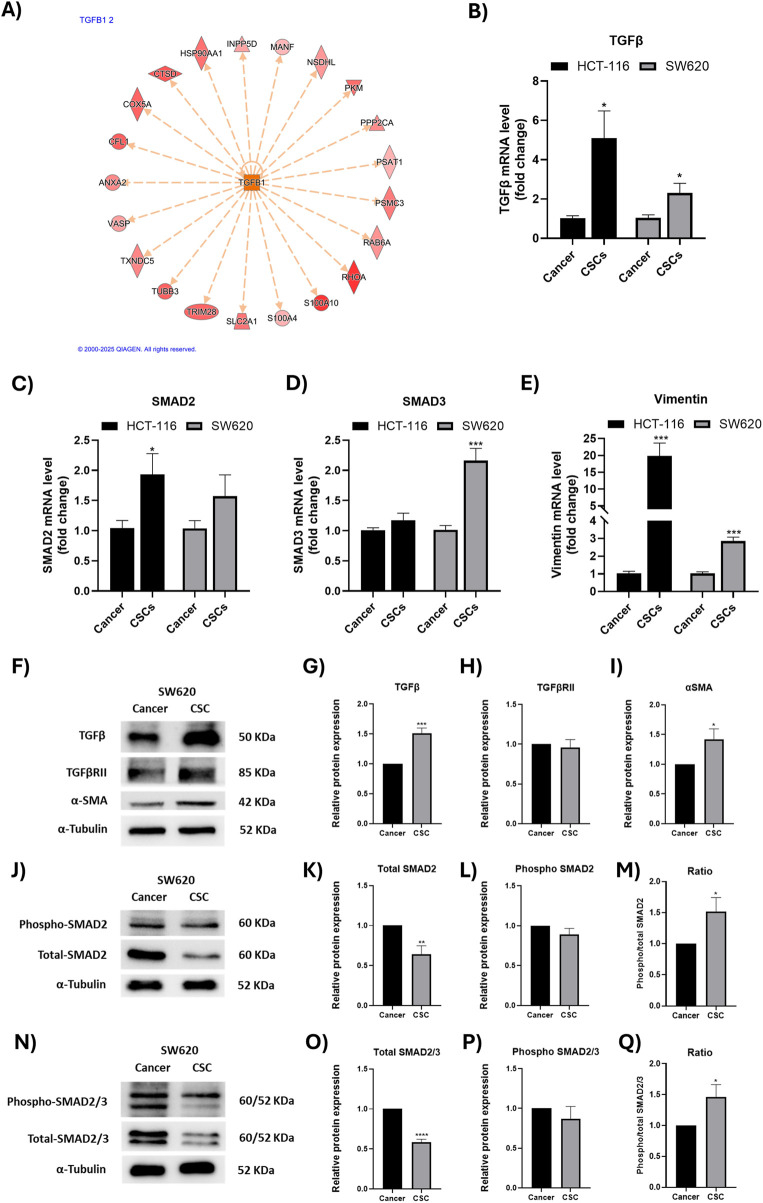
Enhanced TGF-β signaling in colorectal CSC-enriched spheroids. **(A)** Ingenuity Pathway Analysis (IPA) network identifying TGF-β1 as a predicted activated upstream regulator in SW620 CSC-enriched spheroids. The network displays differentially abundant downstream target proteins detected by LC-MS/MS, with red color intensity indicating increased abundance in CSCs relative to bulk cancer cells. Dashed orange arrows represent predicted indirect activation relationships inferred by IPA based on curated knowledge. **(B–E)** RT-qPCR validation of selected TGF-β/SMAD pathway-related genes in SW620 and HCT-116 CSC-enriched spheroids compared with their corresponding bulk cancer cells. mRNA levels were normalized to GAPDH and are presented as fold change relative to bulk cancer cells within the same cell line. **(F,J,N)** Representative immunoblots of key TGF-β pathway mediators in SW620 CSC-enriched spheroids and corresponding bulk cancer cells, using α-tubulin as a loading control. (**(G-I)**, **(K-M)**, **(O-Q)**) Densitometric quantification of protein levels, expressed as relative protein abundance in CSCs versus bulk cancer cells. Data are presented as mean ± SEM from at least three independent experiments. *P < 0.05, **P < 0.01, ***P < 0.001, ****P < 0.0001.

TGF-β was identified among the activated upstream regulators by IPA in CSC-enriched spheroids derived from both SW620 and HCT-116 colorectal cancer cell lines (Figures 4A,B). The predicted activation of TGF-β signaling was supported by the coordinated upregulation of multiple downstream target proteins, including S100A4, SLC2A1, INPP5D, PKM, RHOA, and PSAT1, as illustrated in the IPA regulatory network (Figure 5A). In parallel, several canonical and non-canonical mediators linked to TGF-β signaling were also predicted to be activated in CSCs, including SMAD3, MYC, HIF-1-related signaling.

The proteomic analysis and pathway-level predictions of TGF-β were further validated at the mRNA level. RT-qPCR analysis demonstrated a significant increase in *TGFB* mRNA expression, together with elevated levels of its downstream canonical mediators (*SMAD2* and *SMAD3*), in CSC-enriched spheroids (Figures 5B–D). Moreover, the CSCs of both cell lines exhibited a marked upregulation of *vimentin*, a key epithelial-mesenchymal transition (EMT) marker (Figure 5E). To further validate these findings at the protein level, expression of key TGF-β signaling components was further assessed by Western blot analysis in SW620 cells. As shown in Figures 5F–Q, TGF-β was upregulated in CSCs relative to bulk cancer cells, in agreement with both proteomic and mRNA expression data, which was accompanied by increased expression of the downstream target α-smooth muscle actin (α-SMA). On the other hand, while the total SMAD2 and SMAD3 expression were reduced in CSCs, the phospho-to-total SMAD2 and SMAD2/3 ratio increased. Notably, TGF-βRII expression did not differ between CSCs and bulk cells. Collectively, these findings indicate higher activation of TGF-β signaling in CSCs compared with bulk cancer cells.

## 4 Discussion

Despite advances in CRC therapy, resistance and relapse remain major clinical challenges (Dekker et al., 2019). Accumulating evidence indicates that these adverse outcomes are driven, at least in part, by CSCs (Jiang et al., 2025; Chu et al., 2024), which exhibit pronounced phenotypic plasticity and dynamic interactions with the tumor microenvironment (Rasool et al., 2019; Maccalli et al., 2019; Tomei et al., 2021; Maccalli et al., 2017). Understanding the molecular programs underlying these CSC states is therefore critical for improving therapeutic durability.

In our recent work, we enriched putative CSC populations from CRC cell lines and confirmed upregulation of stemness markers. These CSC-enriched spheroids also exhibited increased expression of inhibitory immune checkpoints (e.g., PD-L1, B7-H3, and CD47), suggesting a role in immune modulation (Hussein et al., 2026). Nevertheless, the molecular alterations underlying these phenotypic features remain incompletely understood. For this purpose, mass spectrometry-based label-free proteomic approach was utilized to compare colorectal CSC-enriched spheroids with their corresponding parental bulk cancer cells, aiming to identify potential novel CSC biomarkers and deepen the understanding of CSC-associated molecular programs, with a focus on immunomodulatory features.

Proteomic profiling of CSC-enriched spheroids from two distinct CRC cell lines (HCT-116 and SW620) revealed convergent pathway- and function-level alterations, despite differences in individual protein abundance, supporting the presence of heterogeneous CSC subpopulations that nonetheless share core CSC-associated signatures. Pathways related to metabolic reprogramming, enhanced invasion/EMT, hypoxia adaptation, and immunomodulatory features were frequently predicted to be activated, whereas tumor-suppressive programs were frequently predicted to be inhibited.

For instance, HMGCS1, CTSD, and CORO1B were consistently upregulated at the proteomic level in CSC-enriched spheroids from both cell lines and showed a generally consistent trend at the mRNA level. Notably, we found that HMGCS1, a key upstream enzyme in the mevalonate pathway, is among the markedly elevated markers in our colorectal CSC datasets, with magnitudes exceeding those reported for several commonly used stemness-associated markers (Hussein et al., 2026; Zhou et al., 2016; Abbas et al., 2023). HMGCS1 has been implicated as a mediator of stemness in breast cancer, where its knockdown reduced the CSC fraction (Walsh et al., 2020; Lee et al., 2016). Similarly, in gastric cancer, HMGCS1 has been reported to elevate the expression of pluripotency genes (OCT4 and SOX2), enhance tumor-sphere formation, and promote *in vivo* tumor growth and metastatic potential (Wang et al., 2020). Collectively, these findings support HMGCS1 as a putative regulator of CSCs and as an attractive therapeutic target. Nevertheless, further functional studies in colorectal CSC models are needed to clarify how HMGCS1 and the mevalonate axis contribute to CSC maintenance and related phenotypes.

Metabolic reprogramming has emerged as a hallmark of CSCs; however, the literature reports conflicting findings regarding the precise metabolic alterations observed across different CSC subpopulations (Yadav et al., 2020; Yasuda et al., 2021). For example, several studies report that CSCs preferentially rely on glycolysis rather than mitochondrial oxidative phosphorylation (OXPHOS), exhibiting higher glycolytic activity than bulk cancer cells to support the maintenance of stemness (Zhou et al., 2011). On the other hand, other reports show that distinct CSC subsets depend primarily on mitochondrial OXPHOS for energy production (Vlashi et al., 2011). In line with this, our proteomic analyses revealed enrichment of multiple metabolic pathways in CSC-enriched spheroids from both cell lines. Notably, CSCs exhibited prominent alterations in cholesterol biosynthesis and related lipid metabolic pathways, together with changes in glucose metabolism and oxidative phosphorylation. These metabolic shifts were accompanied by predicted activation of hypoxia-associated signaling, including HIF signaling, along with upregulation of HIF-1α and EPAS1 (HIF-2α) genes. Collectively, these coordinated metabolic and hypoxia-linked alterations may facilitate CSC adaptation to different tissue contexts and various microenvironmental pressures, thereby conferring a selective survival advantage (Lee H. et al., 2025).

MLXIPL, also known as carbohydrate-responsive element-binding protein (ChREBP), is a key transcriptional regulator of gluco-lipid metabolism, and has been reported to function as an oncogene in colon cancer and hepatocellular carcinoma, whereas its inhibition was shown to suppress tumor growth (Dong et al., 2021; Benichou et al., 2024; Lei et al., 2020). MLXIPL, is another marker that was highly elevated at the mRNA level in our CSC-enriched spheroids. Although MLXIPL was not directly detected in the proteomic dataset, IPA predicted MLXIPL among the top upstream regulators in CSCs. In contrast, Zheng et al. reported that MLXIPL may act as a tumor suppressor in acute myeloid leukemia, where it promoted leukemia cell differentiation (Zeng et al., 2016). Notably, while MLXIPL has been linked to metabolic reprogramming and aggressive tumor phenotypes, its direct functional role in maintaining CSCs has not been evaluated. Therefore, more studies are needed to delineate its potential tissue-specific functions and its contribution to CSC-associated stemness programs.

EMT is closely interconnected with CSCs, collaboratively promoting cancer cell plasticity, metastatic ability, and therapeutic resistance (Jiang et al., 2025). Consistently, IPA analysis predicted activation of functions related to cell movement, migration, and invasion in CSC-enriched spheroids. These predictions were supported by the differential abundance of multiple proteins previously implicated in pro-invasive processes (e.g., Vimentin, TMSB4X, RHOA, S100A10, GNB1, CBX3, RPSA, and EEF1A1). These findings highlight potentially novel contributors to CSC-associated EMT and invasion. Moreover, these features were accompanied by predicted activation of EMT- and invasion-related pathways, including Notch (Shi et al., 2024), HIF-1α (Shen et al., 2024), TGF-β (Xu et al., 2009), Rho Family GTPases (Clayton and Ridley, 2020), integrin (Pang et al., 2023), and VEGF (Lee C. et al., 2025) signaling.

With the expanding clinical application of cancer immunotherapy, research has increasingly focused on defining tumor immune-evasion mechanisms, including those driven by CSCs (Rasool et al., 2019; Maccalli et al., 2019). Our proteomic analysis revealed upregulation of several proteins previously implicated in immune suppression, including PRKCSH (Xiyuan et al., 2025), ITGB4 (Lin et al., 2026), PKM (Chen et al., 2021), CDC42 (Jiang et al., 2023), and RHOA (Koo et al., 2025) in CSC-enriched spheroids, supporting a role for CSCs in shaping an immune-evasive phenotype.

Interestingly, our data highlight TGF-β1 as a predicted activated upstream regulator in CSC-enriched spheroids. In the normal intestinal epithelium, TGF-β acts as a tumor suppressor by inhibiting cell proliferation and inducing apoptosis (Derynck et al., 2021). During CRC progression, many tumors evade TGF-β-mediated growth inhibition, allowing TGF-β signaling to shift toward pro-tumorigenic functions, characterized by enhanced EMT signaling, invasion, and therapeutic resistance (Luo et al., 2025). Moreover, TGF-β signaling has been directly linked to suppression of antitumor immunity and conferring resistance to adaptive cell transfer and ICP blockade immunotherapies (Miao et al., 2019; Xu et al., 2025; Mariathasan et al., 2018). Although dysregulated TGF-β signaling has been repeatedly linked to stemness, the molecular determinants governing this switch in CSCs remain incompletely defined (Chruścik et al., 2018; Itatani et al., 2019; Di Tomaso et al., 2010). A recent multi-omic analysis showed enrichment of TGF-β pathway in primary colorectal CSCs, which was attenuated following transfection with miR-15a and miR-196a mimics (Tout et al., 2025). This miRNA-mediated inhibition of TGF-β signaling was associated with reduced CSC proliferation *in vitro* and decreased tumorigenicity and migration in a zebrafish xenograft model (Tout et al., 2025). Consistently, our proteomic profiling and validation by RT-qPCR and immunoblotting demonstrated upregulation of TGF-β and downstream targets (i.e., α-SMA, vimentin) in CSC-enriched spheroids. Nevertheless, the total SMAD2/3 protein levels were reduced despite a trend toward increased SMAD2/3 mRNA expression, suggesting post-transcriptional regulation of SMAD abundance (Zhang J. et al., 2014). Under sustained TGF-β signaling, activated SMAD2 and SMAD3 can be ubiquitinated and targeted for proteasome-mediated degradation, which may contribute to reduced total SMAD abundance despite ongoing pathway engagement (Gao et al., 2009). Additionally, CSC-associated TGF-β effects may be mediated through non-canonical (Smad-independent) signaling, including the PI3K-AKT-mTOR, ERK, and p38 MAPK pathways and Rho GTPases (Deng et al., 2024). Although canonical TGF-β signaling can repress MYC transcription, we observed increased c-MYC together with elevated HIF-1α expression in SW620 CSC-enriched spheroids, suggesting attenuation of the tumor-suppressive arm of TGF-β signaling in CSCs. In this line, Huang et al. showed that under hypoxia, HIF-1α binds phosphorylated SMAD3 and switches its function, resulting in increased c-MYC expression (Huang et al., 2021). The enrichment of immunomodulatory markers, together with the identification of TGF-β1 as a predicted activated upstream regulator in CSC-enriched spheroids, provides a rationale for investigating TGF-β-directed strategies, including rational combinations such as TGF-β inhibition with immune checkpoint blockade, particularly in CSC-rich contexts.

Despite providing novel insights into the proteomic landscape of colorectal CSCs, several limitations should be acknowledged. First, our findings were derived from established CRC cell lines cultured under spheroid conditions. Although this approach is widely employed for CSC enrichment, it may not fully capture the heterogeneity and microenvironmental complexity of primary colorectal CSCs. Therefore, future studies should validate the identified differentially abundant proteins and pathway alterations in primary CSC populations isolated from patient-derived tumors to strengthen translational relevance. Second, although key targets and upstream regulators (e.g., HMGCS1, MLXIPL, and TGF-β signaling components) were validated at the mRNA and/or protein levels, functional validation of the differentially abundant proteins and predicted pathways was beyond the scope of this study. Future loss- and gain-of-function studies and *in vivo* assays will be necessary to determine their causal roles in CSC maintenance, metabolic reprogramming, immune modulation, and therapeutic resistance. In addition, future proteomic studies should incorporate PTM-focused analyses (e.g., phosphoproteomics and acetylomics) to reveal regulatory mechanisms beyond global protein abundance.

In conclusion, our mass spectrometry-based proteomic profiling of colorectal CSC-enriched spheroids revealed proteomic changes and predicted pathway alterations related to metabolic reprogramming, hypoxia adaptation, EMT/invasion programs, and immunomodulatory signaling. This analysis highlights several potential mediators linked to CSC-associated phenotypes (e.g., HMGCS1, MLXIPL, PRKCSH, ITGB4, TGF-β) that, upon further functional validation, may serve as potential stemness biomarkers or therapeutic targets. However, additional mechanistic studies are needed to define their functional role. Moreover, because these findings were derived from CSC-enriched spheroids generated from CRC cell lines, future validation in primary colorectal CSCs and patient cohorts will be important to strengthen translational relevance.

## Data Availability

The mass spectrometry proteomics data have been deposited to the ProteomeXchange Consortium via the PRIDE partner repository with the dataset identifier PXD075150.
