# Supplementary material for: Comparative proteomic analysis of colorectal cancer stem cells reveals potential biomarkers and altered pathways
Source: Front Mol Biosci. 2026 Apr 20;13:1801265. doi: 10.3389/fmolb.2026.1801265 (PMC13135944; doi:10.3389/fmolb.2026.1801265)
Supplement: Supplementary file 1 [file Supplementaryfile1.docx]

Supplementary Material

# Supplementary Tables

**Table S1 RT-qPCR primers used in the present study**

| Gene | Primer sequences (5’-3’) |
| --- | --- |
| HMGCS1 | Forward primer: TGTACACATCTTCAGTATATGGTTCCC |
|  | Reverse primer: AAGAAAACACTCCAATTCTCTTCCCT |
| NRAS | Forward primer: CCAATACATGAGGACAGGCGA |
|  | Reverse primer: TCACACTTGTTTCCCACTAGCA |
| CORO1B | Forward primer: TCACAGAGGAGCCTCCCTAC |
|  | Reverse primer: GAGGTCCGACTTTCTTGGCA |
| CUTA | Forward primer: CCTGCGTCAACCTCATCCC |
|  | Reverse primer: GCAATTACCTCGGCCACTTC |
| PRKCSH | Forward primer: CCCGCAACAAGTTCGAGGA |
|  | Reverse primer: AGTTTGGGTTTCTGCGAGACAAG |
| PCNA | Forward primer: CAAGTAATGTCGATAAAGAGGAGG |
|  | Reverse primer: GTGTCACCGTTGAAGAGAGTGG |
| ITGB4 | Forward primer: GCTTCACACCTATTTCCCTGTC |
|  | Reverse primer: GACCCAGTCCTCGTCTTCTG |
| PKM | Forward primer: ATGTCGAAGCCCCATAGTGAA |
|  | Reverse primer: TGGGTGGTGAATCAATGTCCA |
| CDC42 | Forward primer: CTGTCAAGTATGTGGAGTGTTCTGC |
|  | Reverse primer: CTCTTCTTCGGTTCTGGAGGCT |
| RHOA | Forward primer: TCTGTCCCAACGTGCCCATCAT |
|  | Reverse primer: CTGCCTTCTTCAGGTTTCACCG |
| CTSD | Forward primer: CAGAAGCTGGTGGACCAGAAC |
|  | Reverse primer: TGCGGGTGACATTCAGGTAG |
| MYC | Forward primer: GCTGCTTAGACGCTGGATTT |
|  | Reverse primer: CGAGGTCATAGTTCCTGTTGG |
| MLXIPL | Forward primer: GCAGTATCGACCCCACAC |
|  | Reverse primer: TCCAGATGGCGTTGTTCA |
| EGF | Forward primer: TGTCCACGCAATGTGTCTGAA |
|  | Reverse primer: CATTATCGGGTGAGGAACAACC |
| VEGFA | Forward primer: AGGGCAGAATCATCACGAAGT |
|  | Reverse primer: AGGGTCTCGATTGGATGGCA |
| HIF-1α | Forward primer: TTTTGGCAGCAACGACACAG |
|  | Reverse primer: GCGGTGGGTAATGGAGACAT |
| HIF-2 | Forward primer: TCCACCTTCAAGACAAGGTCTG |
|  | Reverse primer: GTACATTTGCGCTCAGTGGC |
| TGFβ1 | Forward primer: TACAGCAACAATTCCTGGCG |
|  | Reverse primer: AAGCCCTCAATTTCCCCTCC |
| SMAD2 | Forward primer: ACTAACTTCCCAGCAGGAAT |
|  | Reverse primer: GTTGGTCACTTGTTTCTCCA |
| SMAD3 | Forward primer: GCCTGTGCTGGAACATCATC |
|  | Reverse primer: TTGCCCTCATGTGTGCTCTT |
| Vimentin | Forward primer: TGTCCAAATCGATGTGGATGTTTC |
|  | Reverse primer: TTGTACCATTCTTCTGCCTCCTG |

Table S2 List of primary and secondary antibodies used in this study for Western blot (WB)

| Antibody | Clone | Host/Isotype | Company | Catalog Number | Dilution | Molecular Weight |
| --- | --- | --- | --- | --- | --- | --- |
| TGFβ | Polyclonal | Rabbit IgG | Cell Signaling | #3711 & #77397 | 1:1000 | 12, 25, 45 to 65 |
| TGFβRII | E5M6F | Rabbit IgG | Cell Signaling | #77397 | 1:1000 | 85 KDa |
| Phospho-SMAD2 (Ser465/Ser467) | E8F3R | Rabbit IgG | Cell Signaling | #77397 | 1:1000 | 60 KDa |
| SMAD2 | D43B4 | Rabbit IgG | Cell Signaling | #77397 | 1:1000 | 60 KDa |
| Phospho-SMAD2 (Ser465/467)/ SMAD3 (Ser423/425) | D27F4 | Rabbit IgG | Cell Signaling | #77397 | 1:1000 | 52, 60 KDa |
| SMAD 2/3 | D7G7 | Rabbit IgG | Cell Signaling | #77397 | 1:1000 | 52, 60 KDa |
| α-SMA | D4K9N | Rabbit IgG | Cell Signaling | #77397 | 1:1000 | 42 KDa |
| α-Tubulin (HRP Conjugate) | DM1A | Mouse IgG1 | Cell Signaling | 12351 | 1:1000 | 52 kDa |
| Anti-mouse IgG, HRP-linked | Polyclonal | Horse | Cell Signaling | 7076 | 1:1000-1:3000 | N/A |
| Anti-rabbit IgG, HRP-linked Antibody | Polyclonal | Goat | Cell Signaling | 7074 | 1:1000-1:3000 | N/A |

Table S3 List of differentially expressed proteins in SW620 CSC-enriched spheroids linked to cell death of tumor cells by IPA

| Protein in dataset | Prediction  (based on measurement direction) | Expr Log Ratio | Findings |
| --- | --- | --- | --- |
| RPL27A | Decreased | 5.42 | Decreases (1) |
| EEF1A1 | Decreased | 4.831 | Decreases (1) |
| SOD1 | Affected | 4.794 | Affects (1) |
| SF3B2 | Decreased | 4.672 | Decreases (1) |
| VDAC2 | Decreased | 4.305 | Decreases (1) |
| RPS5 | Decreased | 4.224 | Decreases (1) |
| RPL13 | Decreased | 4.163 | Decreases (1) |
| RPL9 | Decreased | 4.08 | Decreases (1) |
| RPS13 | Decreased | 4.07 | Decreases (1) |
| RPS18 | Decreased | 3.699 | Decreases (1) |
| RPS3A | Affected | 3.693 | Affects (2) |
| CDC42 | Decreased | 3.666 | Decreases (1) |
| RPS15A | Decreased | 3.402 | Decreases (1) |
| RPL23A | Decreased | 3.391 | Decreases (1) |
| U2AF1 | Decreased | 3.362 | Decreases (1) |
| ST13 | Increased | 3.346 | Increases (1) |
| RPS28 | Decreased | 3.321 | Decreases (1) |
| RPS21 | Decreased | 3.255 | Decreases (1) |
| RPS3 | Decreased | 3.246 | Decreases (1) |
| PSMC5 | Decreased | 3.089 | Decreases (1) |
| PPID | Affected | 3.07 | Affects (1) |
| AIFM1 | Increased | 3.068 | Increases (1) |
| ANXA2 | Decreased | 3.009 | Decreases (2) |
| PSMD11 | Decreased | 2.951 | Decreases (1) |
| PPP2CA | Increased | 2.93 | Increases (1) |
| PUF60 | Decreased | 2.854 | Decreases (1) |
| MAPK14 | Decreased | 2.702 | Decreases (1) |
| FADD | Increased | 2.698 | Increases (4) |

Table S4 List of differentially expressed proteins in SW620 CSC-enriched spheroids linked to metabolism of macromolecules by IPA

| Protein in dataset | Prediction  (based on measurement direction) | Expr Log Ratio | Findings |
| --- | --- | --- | --- |
| SUB1 | Decreased | 6.529 | Decreases (3) |
| PCNA | Increased | 5.589 | Increases (15) |
| RPL27A | Affected | 5.42 | Affects (2) |
| RHOA | Affected | 5.254 | Affects (129) |
| EEF1A1 | Increased | 4.831 | Increases (31) |
| SOD1 | Decreased | 4.794 | Decreases (248) |
| GORASP2 | Affected | 4.365 | Affects (2) |
| GSN | Increased | 4.289 | Increases (39) |
| CTSD | Increased | 4.262 | Increases (47) |
| CDC37 | Increased | 4.258 | Increases (40) |
| PITRM1 | Increased | 4.238 | Increases (10) |
| RPS5 | Affected | 4.224 | Affects (5) |
| SUGT1 | Affected | 4.126 | Affects (1) |
| CFL1 | Increased | 4.051 | Increases (8) |
| LAMP1 | Affected | 3.919 | Affects (3) |
| TRIM28 | Affected | 3.903 | Affects (16) |
| ANXA3 | Increased | 3.87 | Increases (1) |
| PGK1 | Increased | 3.861 | Increases (10) |
| CPNE1 | Affected | 3.729 | Affects (1) |
| PKM | Increased | 3.706 | Increases (152) |
| CAPN1 | Increased | 3.694 | Increases (119) |
| RPS3A | Increased | 3.693 | Increases (3) |
| CDC42 | Increased | 3.666 | Increases (57) |
| HSP90AA1 | Increased | 3.568 | Increases (45) |
| NACA | Increased | 3.543 | Increases (4) |
| RPS15A | Increased | 3.402 | Increases (10) |
| PRPF19 | Increased | 3.399 | Increases (17) |
| CACYBP | Increased | 3.365 | Increases (11) |
| U2AF1 | Affected | 3.362 | Affects (12) |
| DPP3 | Affected | 3.359 | Affects (12) |
| RPS28 | Affected | 3.321 | Affects (4) |
| RPS3 | Decreased | 3.246 | Decreases (2) |
| TFAM | Increased | 3.14 | Increases (18) |
| RPL22 | Decreased | 3.095 | Decreases (6) |
| PSMC5 | Affected | 3.089 | Affects (10) |
| AIFM1 | Increased | 3.068 | Increases (42) |
| PSMD11 | Affected | 2.951 | Affects (1) |
| ERP44 | Increased | 2.944 | Increases (2) |
| PPP2CA | Affected | 2.93 | Affects (109) |
| NPEPPS | Increased | 2.8 | Increases (8) |
| CCT6A | Affected | 2.759 | Affects (27) |
| FADD | Increased | 2.698 | Increases (70) |
| SARS1 | Affected | 2.693 | Affects (2) |
| RPS9 | Increased | 2.622 | Increases (3) |
| SKP1 | Affected | 2.593 | Affects (6) |
| RAN | Affected | 2.576 | Affects (2) |
| ASPH | Increased | 2.55 | Increases (17) |
| EPRS1 | Decreased | 2.503 | Decreases (43) |
| ATG4B | Increased | 2.486 | Increases (13) |
| TF | Affected | 2.446 | Affects (26) |
| NRAS | Increased | 2.431 | Increases (91) |
| TUFM | Affected | 2.4 | Affects (1) |
| VPS4A | Increased | 2.257 | Increases (3) |
| RRBP1 | Increased | 2.195 | Increases (5) |
| CUL4B | Affected | 2.151 | Affects (38) |
| NDUFA13 | Increased | 2.142 | Increases (35) |
| NCKAP1 | Affected | 2.098 | Affects (4) |
| ABCB7 | Increased | 2.075 | Increases (12) |
| HDAC2 | Increased | 2.069 | Increases (29) |
| PSMC2 | Affected | 2.065 | Affects (1) |
| TARDBP | Affected | 2.058 | Affects (65) |
| S100A4 | Affected | 1.983 | Affects (61) |
| LSS | Affected | 1.964 | Affects (4) |
| RPS20 | Increased | 1.83 | Increases (14) |
| PPM1G | Decreased | 1.813 | Decreases (4) |
| PPIB | Increased | 1.641 | Increases (6) |
| ADD1 | Affected | 1.633 | Affects (4) |
| EIF3K | Affected | 1.586 | Affects (2) |
| ARHGEF1 | Decreased | 1.547 | Decreases (5) |
| EXOSC4 | Affected | -1.857 | Affects (2) |
| THOP1 | Affected | -2.041 | Affects (19) |

Table S5 List of differentially expressed proteins in SW620 CSC-enriched spheroids linked to invasion of cells by IPA

| Protein in dataset | Prediction  (based on measurement direction) | Expr Log Ratio | Findings |
| --- | --- | --- | --- |
| TMSB4X | Increased | 5.478 | Increases (1) |
| RHOA | Increased | 5.254 | Increases (51) |
| S100A10 | Increased | 5.235 | Increases (8) |
| GNB1 | Increased | 5.035 | Increases (4) |
| CBX3 | Increased | 5.012 | Increases (8) |
| RPSA | Increased | 4.967 | Increases (1) |
| EEF1A1 | Increased | 4.831 | Increases (5) |
| STMN1 | Increased | 4.689 | Increases (11) |
| SF3B2 | Increased | 4.672 | Increases (1) |
| HMGCS1 | Increased | 4.333 | Increases (2) |
| ALDOA | Increased | 4.297 | Increases (8) |
| GSN | Increased | 4.289 | Increases (20) |
| CTSD | Affected | 4.262 | Affects (1) |
| DPYSL3 | Decreased | 4.087 | Decreases (2) |
| CFL1 | Affected | 4.051 | Affects (1) |
| PA2G4 | Increased | 4.048 | Increases (5) |
| TUBB3 | Increased | 4.034 | Increases (1) |
| EEF1D | Increased | 3.999 | Increases (1) |
| PGK1 | Increased | 3.861 | Increases (2) |
| DPYSL2 | Decreased | 3.837 | Decreases (12) |
| CPNE1 | Increased | 3.729 | Increases (3) |
| PKM | Increased | 3.706 | Increases (16) |
| CDC42 | Increased | 3.666 | Increases (25) |
| HSP90AA1 | Increased | 3.568 | Increases (11) |
| SLC2A1 | Increased | 3.513 | Increases (8) |
| FKBP4 | Increased | 3.469 | Increases (4) |
| RPS15A | Increased | 3.402 | Increases (2) |
| PRPF19 | Increased | 3.399 | Increases (6) |
| PDIA4 | Decreased | 3.315 | Decreases (6) |
| PCMT1 | Increased | 3.297 | Increases (8) |
| ACSL3 | Decreased | 3.214 | Decreases (2) |
| ACSL4 | Increased | 3.111 | Increases (2) |
| FKBP11 | Increased | 3.046 | Increases (2) |
| ANXA2 | Increased | 3.009 | Increases (6) |
| FDFT1 | Decreased | 2.99 | Decreases (1) |
| PTPN12 | Affected | 2.955 | Affects (1) |
| LRRC59 | Increased | 2.927 | Increases (2) |
| AKAP12 | Decreased | 2.882 | Decreases (4) |
| PUF60 | Decreased | 2.854 | Decreases (2) |
| G3BP1 | Increased | 2.853 | Increases (6) |
| MLLT11 | Increased | 2.781 | Increases (3) |
| CCT6A | Increased | 2.759 | Increases (6) |
| DNM2 | Increased | 2.742 | Increases (5) |
| AGR2 | Increased | 2.71 | Increases (2) |
| ANXA6 | Increased | 2.703 | Increases (5) |
| MAPK14 | Increased | 2.702 | Increases (5) |
| DBNL | Affected | 2.669 | Affects (4) |
| TIGAR | Increased | 2.649 | Increases (3) |
| ARHGDIA | Decreased | 2.626 | Decreases (3) |
| ASPH | Increased | 2.55 | Increases (5) |
| ASNS | Affected | 2.522 | Affects (1) |
| ATG4B | Increased | 2.486 | Increases (1) |
| NRAS | Increased | 2.431 | Increases (2) |
| VASP | Increased | 2.41 | Increases (1) |
| ABI1 | Decreased | 2.407 | Decreases (2) |
| DYNC1H1 | Increased | 2.36 | Increases (2) |
| COPB2 | Increased | 2.358 | Increases (1) |
| RBBP4 | Increased | 2.292 | Increases (8) |
| ITGB4 | Increased | 2.262 | Increases (15) |
| ARF5 | Decreased | 2.232 | Decreases (1) |
| VPS26A | Increased | 2.207 | Increases (3) |
| G3BP2 | Increased | 2.205 | Increases (5) |
| CUL4B | Increased | 2.151 | Increases (13) |
| NDUFA13 | Decreased | 2.142 | Decreases (1) |
| NCKAP1 | Increased | 2.098 | Increases (3) |
| RPL32 | Increased | 2.096 | Increases (2) |
| TARDBP | Increased | 2.058 | Increases (1) |
| S100A4 | Increased | 1.983 | Increases (9) |
| PSAT1 | Increased | 1.915 | Increases (10) |
| PLOD1 | Increased | 1.893 | Increases (4) |
| MANF | Decreased | 1.858 | Decreases (1) |
| OGDH | Decreased | 1.831 | Decreases (1) |
| PPIB | Increased | 1.641 | Increases (3) |
| VAC14 | Increased | -1.921 | Decreases (2) |

# Supplementary Figures


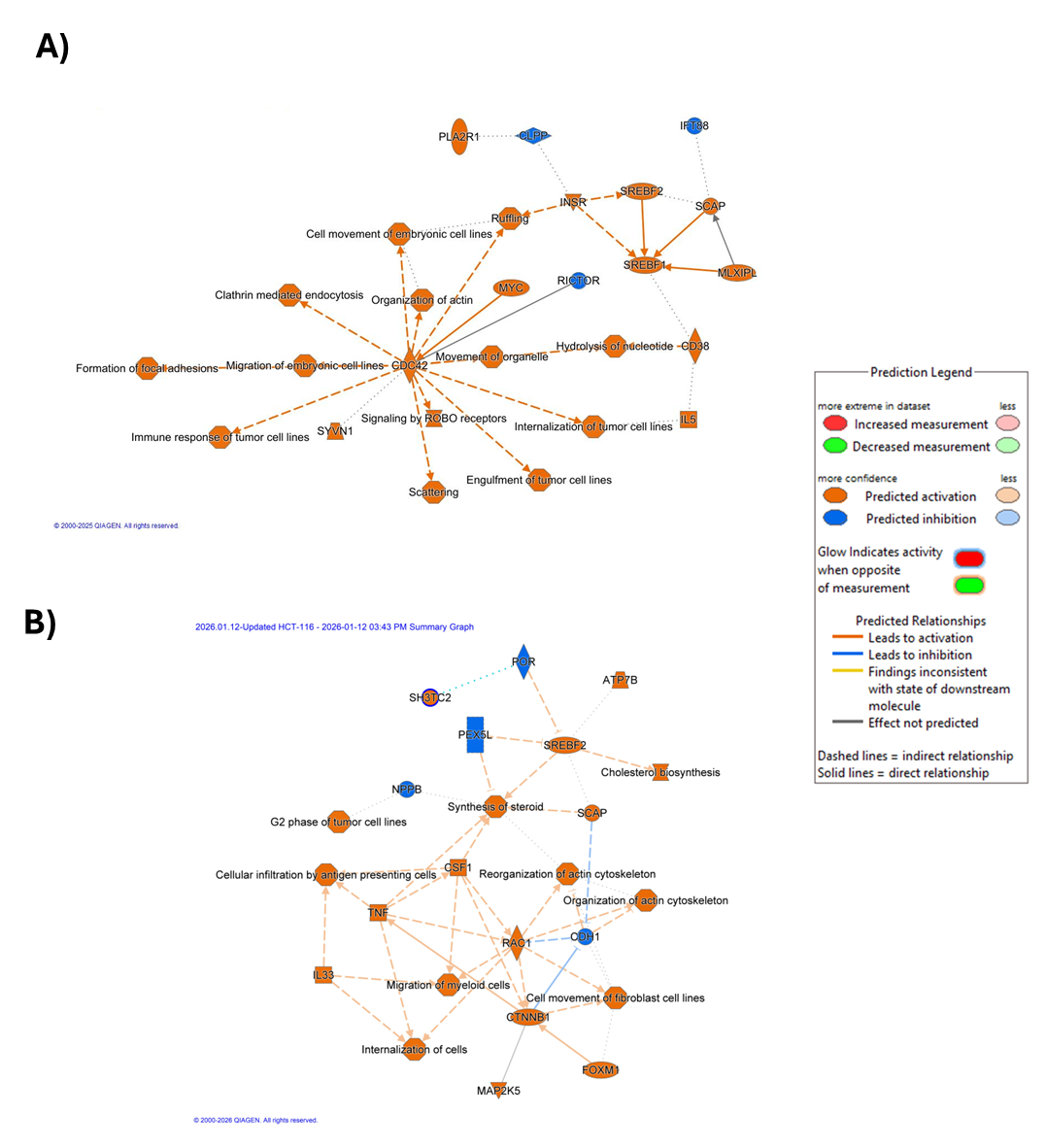


Figure S1. Graphical summary of Ingenuity Pathway Analysis (IPA) results based on differentially expressed proteins in (A) SW620 CSC-enriched spheroids and (B) HCT-116 CSC-enriched spheroids compared with their corresponding bulk (parental) adherent cancer cells.


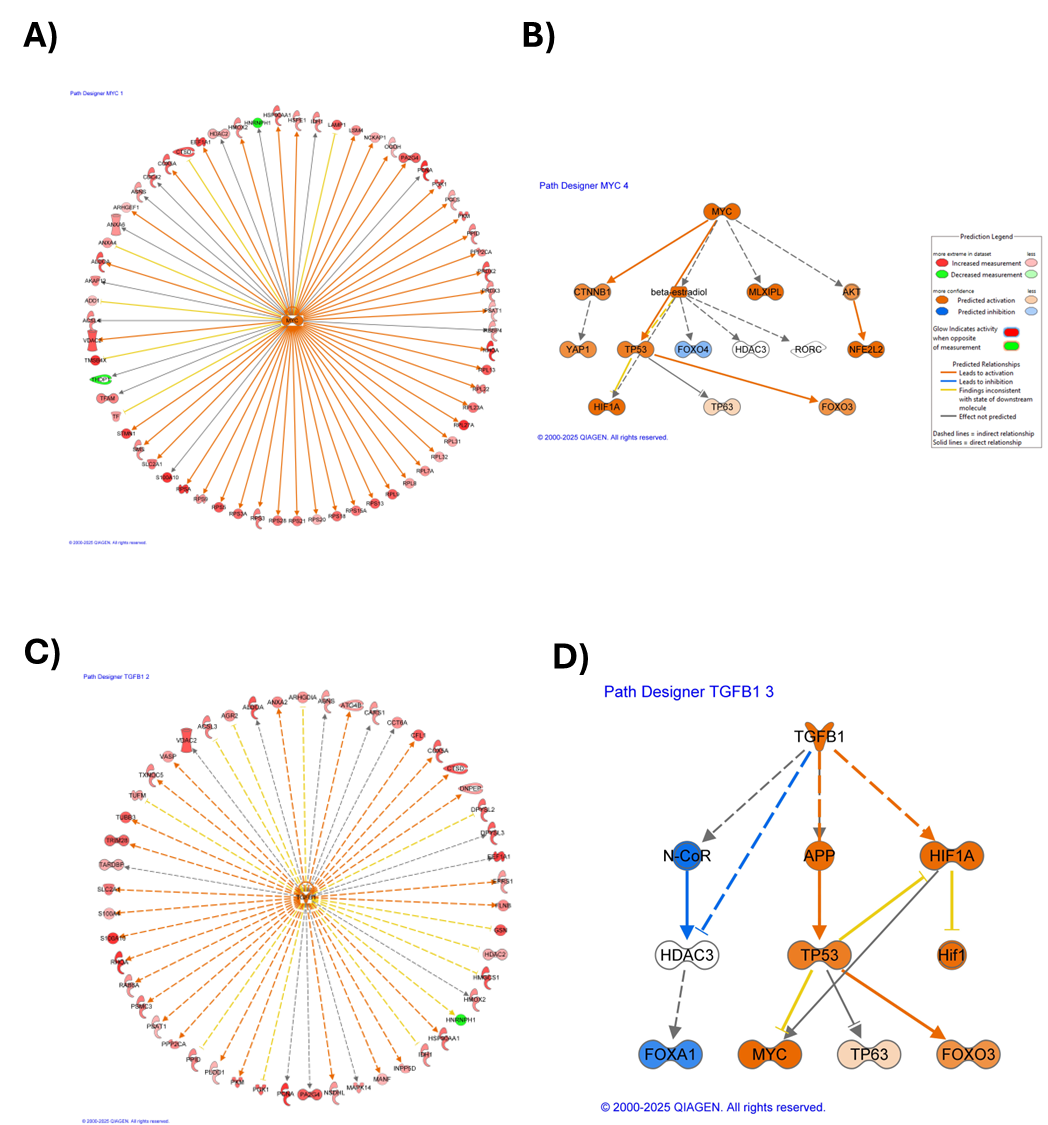


Figure S2. Networks of selected upstream regulators predicted by Ingenuity Pathway Analysis (IPA) in SW620 CSC-enriched spheroids versus parental adherent cells: (A–B) MYC regulator–target and mechanistic networks; (C–D) TGFB1 regulator–target and mechanistic networks.


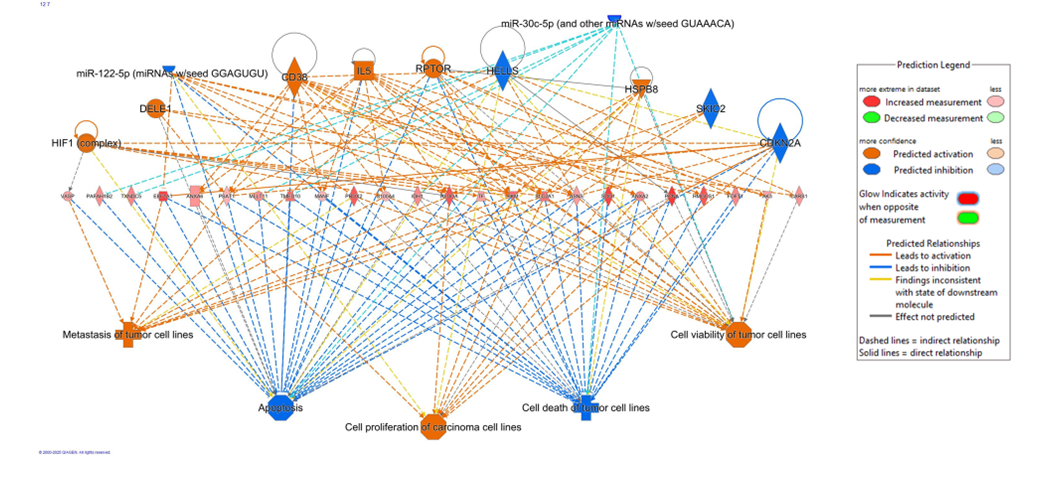


Figure S3. Ingenuity Pathway Analysis (IPA) Regulator Effects network generated from differentially expressed proteins in SW620 CSC-enriched spheroids versus their corresponding parental adherent cells.
